# The effects of myofascial induction therapy in survivors of head and neck cancer: a randomized, controlled clinical trial

**DOI:** 10.1007/s00520-022-07482-9

**Published:** 2022-12-17

**Authors:** Lucía Ortiz-Comino, Lydia Martín-Martín, Noelia Galiano-Castillo, Eduardo Castro-Martín, Miguel Ángel Fernández-Gualda, Mario Lozano-Lozano, Carolina Fernández-Lao

**Affiliations:** 1 Department of Physical Therapy. Health Sciences Faculty, University of Granada, Melilla, Spain; 2 Department of Physical Therapy, Health Sciences Faculty, University of Granada, Granada, Spain; 3 Sport and Health Research Center (IMUDs), Granada, Spain; 4 Unit of Excellence On Exercise and Health (UCEES), University of Granada, Granada, Spain; 5 Instituto de Investigación Biosanitaria Ibs. GRANADA, Granada, Spain

**Keywords:** Head and neck cancer, Survivors, Musculoskeletal manipulations, Manual therapy, Range of motion, Randomized controlled trial

## Abstract

**Purpose:**

We aim to evaluate the effects of myofascial induction therapy (MIT) on the sequelae suffered by the survivors of HNC (sHNC).

**Methods:**

We enrolled 46 sHNC in a randomized controlled trial (RCT), of whom 20 received a MIT protocol and 23 were placed on a waitlist while receiving the recommended treatment for 6 weeks. The MIT protocol included a total of 18 sessions, 3 days a week on alternate days for 6 weeks. Maximal mouth opening, the presence of temporomandibular dysfunction, cervical endurance, active range of motion (AROM), shoulder AROM, handgrip strength, and perceived physical fitness were assessed.

**Results:**

Maximal mouth opening, temporomandibular dysfunction, cervical endurance, and AROM, affected shoulder abduction and unaffected shoulder flexion and external rotation significantly improved (*p* < .05) after an MIT protocol, but only cervical AROM and affected shoulder abduction changes were clinically meaningful. No statistically significant changes were observed in the other shoulder AROM, handgrip strength, or physical fitness perception (*p* > .05).

**Conclusion:**

A 6-week MIT protocol improves mouth opening, TMD, cervical function (endurance and AROM), affected shoulder abduction and unaffected shoulder flexion, and external rotation AROM in the sHNC. However, no changes were observed in most of the shoulder AROM, muscular strength, or perceived physical fitness. Future studies should perform longer follow-up designs, increase the sample size, and include multimodal treatments to address these sequelae in the sHNC.

**Supplementary Information:**

The online version contains supplementary material available at 10.1007/s00520-022-07482-9.

## Introduction

Standard therapy in head and neck cancer (HNC) consists of surgery and radiotherapy, and possibly chemotherapy, all of which often lead to frequent comorbidities [1].

Apart from the pain and reduced mobility that may lead to neck and shoulder dysfunction following surgical treatment [2], one of the most frequent comorbidities is radiation-induced fibrosis. This is caused by damage to the blood vessels responsible for nourishing musculoskeletal tissues and for causing them to become inflamed, which can provoke several complications [3]. Among these, the decreased size of the mouth opening [4], temporomandibular dysfunction (TMD), and an esophageal stricture are the most limiting factors that affect phonation and deglutition. Moreover, muscular tightness and reduced active range of motion (AROM) induced by fibrosis in both the cervical and shoulder regions may appear [5]. Together, these facial, cervical, and shoulder functional impairments along with changes to the physical body, in addition to the changes caused by chemotherapy (e.g., impairments in muscle strength and physical fitness) [6, 7], may negatively influence the quality of life (QoL) of the survivors of HNC (sHNC) [8].

Interventions to ease these sequelae include exercise to improve mouth opening [9] or progressive resistance training of the cervical and shoulder regions [10], as well as manual therapy [11]. This last intervention includes techniques that involve the treatment of soft tissues to improve their mobility and function. The proven benefits of manual therapy include improved circulation, decreased occurrence of muscle spasm, increased AROM, decreased pain, and the release of connective tissue adhesions [12]. Within manual therapy, myofascial induction therapy (MIT) also has several benefits [13]; it consists of a combination of three-dimensional maneuvers involving the different levels of fascial system movement, with the objective of allowing the reestablishment of body balance and reducing painful symptoms to restore normal function to the locomotor apparatus [14]. Through these techniques, the connective tissue is mechanically stimulated, the circulation of antibodies in the fundamental substance is improved [15], the blood supply to the regions with restricted movement is restored, and the correct position of the cells and fibers that compose it is fixed, thus modifying the mechanical changes produced by fibrosis. In this way, the flow of metabolites to and from the fascial tissue is favored, facilitating the recovery process [14].

Due to these characteristics, MIT has been shown to be a possible effective treatment to reduce the sequelae that cancer survivors suffer. At present, the literature analyzing its effects is focused on breast cancer survivors and their sequelae, such as pain and reduced AROM [16–18]. In sHNC, MIT has been investigated for its effectiveness as a treatment for limited mouth opening [19], and a crossover study has recently shown to reduce pain and improve cervical and shoulder AROM after one single application [11], but it is unknown whether a longer protocol of MIT improves TMD, cervical endurance, and shoulder AROM, or if it has some effects on muscular strength or physical fitness. Thus, it is important to develop stronger protocols, such as randomized controlled trials (RCTs), to demonstrate the effects of MIT in sHNC.

The main objective of this study is to evaluate the effects of an MIT protocol on mouth opening, TMD, cervical function (endurance and AROM), and shoulder AROM for sHNC. We also evaluated the effects of this protocol on the muscular strength of the upper extremities and perceived physical fitness. We hypothesize that mouth opening, and cervical function may be positively affected by an MIT protocol compared to the usual care provided to sHNC.

## Methods

We developed a prospective, randomized, controlled, single-blind trial (ClinicalTrials.gov Identifier: NCT04145180) following the CONSORT statement guidelines [20].

### Participants

The participants of this study were sHNC recruited from October 2019 to March 2020 from the Medical Oncology Service of the “Virgen de las Nieves” University Hospital, Granada, Spain. To be eligible for the study, patients had to meet the following criteria: (1) being ≥ 18 years, (2) having finished oncological treatment in the previous 6–24 months, (3) having no metastasis or active cancer, and (4) reporting their specialists TMD, cervical or shoulder impairments related to the oncological treatment. The exclusion criteria were (1) mental or physical illness, (2) previous chronic pain, and (3) previous TMD, cervical, or shoulder impairments.

Participants were randomly allocated (ratio 1:1) into two different groups using computer software (EPIDAT v.4.2, Xunta de Galicia, España). To reassure the blinding of the process, this randomization was performed after the baseline assessment by an external researcher.

Before starting the treatment protocol, three physiotherapists trained in MIT agreed on the applied protocol. All procedures were conducted in accordance with the Declaration of Helsinki of the World Medical Association. Ethical approval for the study was granted by the Biomedical Investigation Ethics Committee, Granada, Spain (CEi-GRANADA Ref: 0045-N-16). All subjects signed the informed consent form to be included in the study.

### Intervention procedure

After the initial assessment, all participants in the intervention group were treated through a physiotherapy protocol (performed by a physiotherapist with extensive experience in MIT) three days a week for 6 weeks, (approximately 40 min each) and performed on alternate days, with an established minimum rest period of 40 h between each session. The potential adverse effects were registered during the intervention program.

Patients were lying in a supine position or sitting in a chair to receive the treatment, depending on the technique applied. Several MIT techniques proposed by Pilat, A. [21] were selected to encompass the intraoral, cervical, and shoulder regions (Supplementary Information 1). The main purpose of this treatment was to relieve fibrosis and fascial restrictions to improve tissue mobility and function. In addition, changes in muscular strength and physical condition were also assessed.

Participants in the control group were on a waitlist and received the usual care recommended by the medical staff during the same period as the intervention group. These recommendations were focused on psychological health, physical activity, and nutritional indications. For ethical reasons, patients in the control group were invited to receive the MIT intervention after their participation in the waitlist.

### Outcome measures

Demographic (age, sex, alcohol consumption, and tobacco habits) and clinical (time since diagnosis, tumor location, tumor stage (t-stage) [22], and medical treatment received) data were recorded at the first appointment with the participants.

The following outcomes were assessed before the first intervention and on the same day after the last intervention by an assessor blinded to the aims of the study and to each patient’s assigned group.

#### Maximal mouth opening (MMO)

Assessed through a sliding caliper, with the patient sitting in a chair. Patients were asked to open their mouths as wide as possible and then the evaluator measured the distance (in mm) between the upper and lower incisors [23]. The reliability of the MMO assessment is excellent, with an intraclass correlation coefficient (ICC) of 0.95–0.96 [24].

#### Temporomandibular dysfunction (TMD)

The Fonseca Anamnestic Index (FAI) was used to assess temporomandibular disorders [25]. This questionnaire with 10 self-administered items has three possible responses that score as follows: yes (10 points), sometimes (5 points), or no (0 points). The total amount is calculated and then categorized into four different scales: (0) no TMD; (1) mild dysfunction; (2) moderate dysfunction, and (3) severe dysfunction. The reliability of the Spanish version of this tool is excellent, with an ICC of 0.93 [26].

#### Cervical muscle endurance

The deep cervical flexor endurance test (DCFET) was used to assess cervical muscle endurance. This test is carried out with the subjects lying in a supine position with the examiner’s hands under their head and first asking them to flex the upper cervical spine and second, maintaining this position, raising the head as little as possible from the examiner’s hands. The time was counted from when the patient raised the head until (1) he or she was not able to maintain the position, (2) the patient’s head rested on the examiner’s hands, or (3) the patient started to feel any pain [27]. The ICC of this test ranges from 0.82 to 0.91 [28].

#### Active Range of motion (AROM)

Cervical AROM was measured in degrees by using a CROM device (Performance Attainment Associates©, Spine Products, Roseville, MN, USA) with the patient sitting with their feet on the floor [29]. The movements assessed were flexion, extension, left and right inclination, and left and right rotation. The ICC ranges from 0.89 to 0.98 depending on the performed movement [30]. Shoulder AROM was bilaterally assessed by a two-arm goniometer with a 360° protractor with the patient lying in the supine position. The movements evaluated were shoulder flexion, abduction, and external and internal rotation. Each movement was recorded once the maximum angle was reached [31]. The ICC was found to be excellent (0.94) [32].

#### Handgrip strength

Determined with a digital dynamometer (TKK 5101 Grip-D; Takey, Tokyo, Japan). Patients started in the standing position, held the dynamometer in the evaluated hand which was extended down the side of their body and were instructed to squeeze it as tightly as possible [33]. Three trials were performed, alternating hands (6 attempts), with a 1-min rest between tests. The mean value of the three attempts was calculated for the data analysis [34]. The reliability of this handgrip dynamometer is very high: its systematic error for test–retest is ≤ 0.3 kg [35].

#### Physical fitness perception

The International Fitness Scale (IFIS) was used [36]. It consists of a self-administered questionnaire that considers the main physical fitness components: cardiorespiratory fitness, muscle strength, agility-speed, and flexibility. Responses are based on a 5-point Likert scale (from “very good” to “very poor”). It has been proven to be a reliable instrument with a 0.54–0.65 coefficient [37].

### Sample size calculation

To detect a minimum of a 5 mm difference in the maximal mouth opening [38] between the groups, with an alpha value of 0.05, and assuming a 95% statistical power, 16 participants per group were needed (i.e., 32 participants in total), assuming a 10% dropout rate. Sample size calculations were performed with G*Power v3.1.9.7 software (Düsseldorf, Germany).

### Statistical analysis

The Kolmogorov–Smirnov test was performed to check the normal distribution of all variables (*p* < 0.05). The baseline differences between groups were analyzed with the *T* test or the Mann–Whitney U test as required, and the chi-squared test or the Fisher’s exact test was used in the case of categorical variables. To examine the changes after the intervention/control period, a 2-way repeated measures analysis of covariance (ANCOVA), or its nonparametric analog test, was performed, with the treatment as the between-group variable and the time (pre-post interventions) as the within-group variable. Sex, age, alcohol consumption, smoking habits, time since diagnosis, and tumor stage were included as covariates. Cohen’s *d* values were calculated to obtain the effect sizes (ES) of the MIT treatment. All *p* values < 0.05 were considered statistically significant. When possible, the obtained results were examined to check if there was a clinically meaningful change. Categorical variables were analyzed with the chi-squared test or the Fisher’s exact test to determine the change score between pre- and postintervention within the intervention or the control group. All statistical analyses were performed per the protocol and using SPSS software v.25.0 (IBM Statistics, Armonk, NY, USA).

## Results

**Fig. 1:**
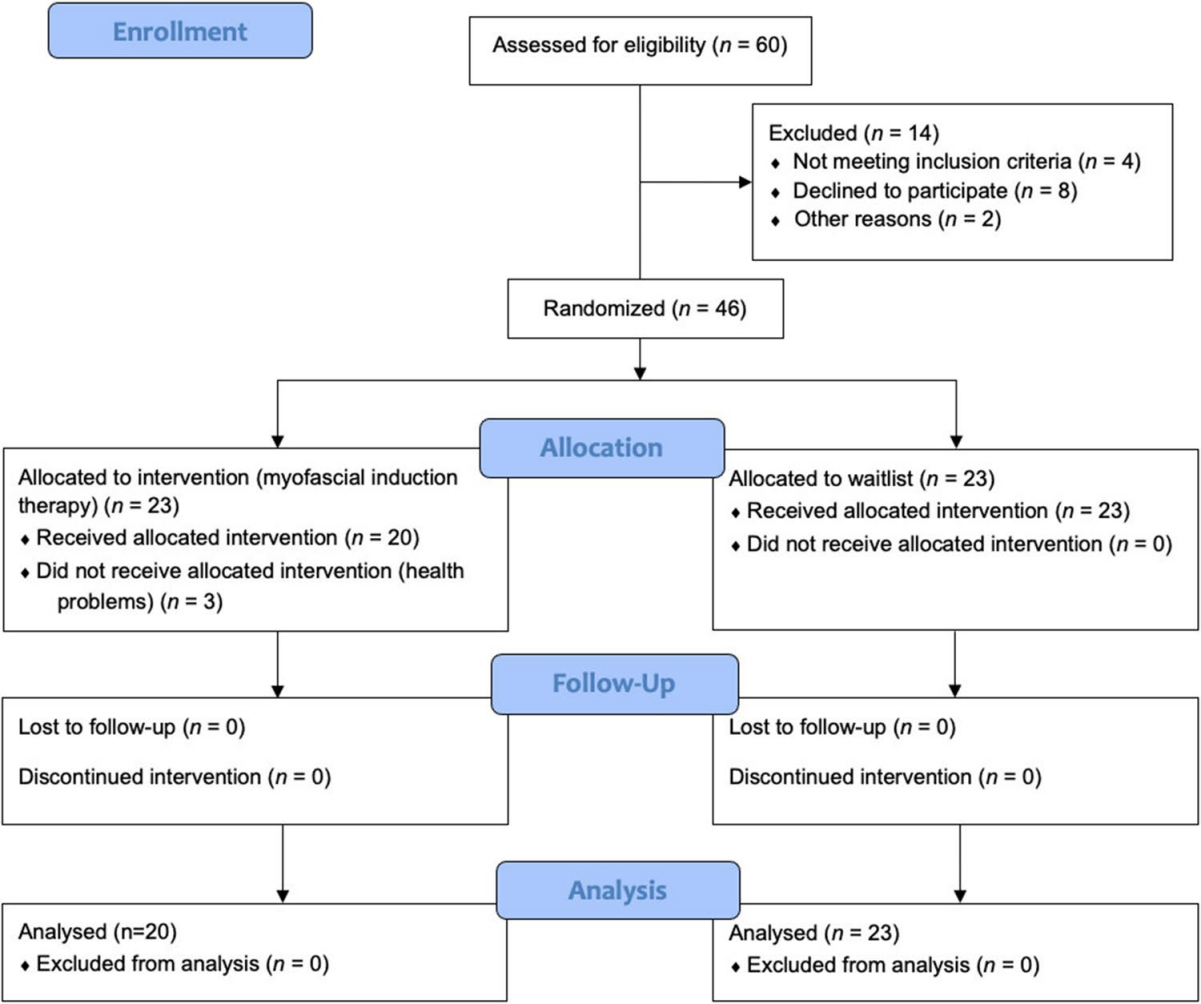
CONSORT flow diagram of participants included on the trial

Because of a high response rate of participation and thus surpassing the sample size required, a total of 46 sHNC were randomized into the two groups, as shown in Fig. 1. Twenty-three participants were allocated to the intervention group and 23 to the control group; however, three participants allocated to the intervention group did not continue with the treatment as they reported health problems; thus, the results of 20 participants were analyzed. No statistically significant differences were observed between the groups at baseline (*p* > 0.05) in any of the demographic and clinical data recorded (Table 1). No adverse effects from the MIT protocol were detected.

**Table 1:** Demographic and clinical data (mean (SD) for continuous data and frequencies (%) for categorical data)

	Intervention group (*n* = 20)	Control group (*n* = 23)	*p* value
Age	59.05 (12.53)	58.22 (11.37)	.820
Gender Male Female	13 (65%) 7 (35%)	18 (78%) 5 (22%)	.334
Alcohol consumption Yes No	11 (55) 9 (45)	12 (52%) 11 (48%)	.853
Smoking habits Non-smoker Ex-smoker Smoker	7 (35%) 11 (55%) 2 (10%)	5 (22%) 15 (65%) 3 (13%)	.624
Time since diagnosis (months)	27.90 (14.46)	24.39 (14.33)	.430
T-stage I II III IV	4 (20%) 4 (20%) 4 (20%) 7 (35%)	4 (17%) 5 (22%) 3 (13%) 9 (38%)	.939
Treatment Surgery Radiotherapy Chemotherapy	13 (65%) 20 (100%) 14 (70%)	12 (52%) 23 (100%) 16 (70%)	.932 N/A .975

T-stage lost data: intervention group (*n* = 1), control group (*n* = 2)

### Maximal mouth opening

ANCOVA revealed a significant improvement in MMO in the intervention group compared with the control group (*F* = 11.871; *p* = 0.001) (Table 2). However, the MMO mean value of the intervention group after the intervention did not surpass the threshold of 35 mm established to differentiate the absence of trismus [4] or the minimal detectable change of 5 mm [38]. The ES of the MIT intervention for this outcome was large (*d* = 1.05; 95% CI 0.4–1.7). When adjusting for covariates, statistical significance did not change.

**Table 2:** Preintervention, postintervention, and change scores for MMO in mm (*n* = 43)

	Intervention group (*n* = 20)	Control group (*n* = 23)	*p* value*	Cohen’s *d*
MMO (mm) Preintervention Postintervention Pre-post change score	30.95 (10.94) 34.87 (11.97) 4.5 (6)	33.39 (14.25) 31.43 (12) − 1.95 (6.07)	*p* = .001	1.05

Values are mean (SD). The mean difference (SD) for pre-post change score. *Time x group interaction (ANCOVA analysis). *ANCOVA* analysis of covariance, *Mm* millimeters, *MMO* maximal mouth opening

### Temporomandibular dysfunction

The chi-squared test found a significant difference (Chi2 = 10.316; *p* = 0.016) for the presence of TMD in the intervention group between the pre- and posttreatment scores, where there were five more patients reporting a lower level of TMD on the FAI, from moderate to mild, and three more patients who reported a higher level of TMD, from moderate to severe (Table 3). No statistically significant changes were observed in the control group (Chi2 = 1.628; *p* = 0.653).

**Table 3:** Fonseca Anamnese Index scores in pre- and post-evaluations and change scores (*n* = 43)

	Intervention group (*n* = 20)			Control group (*n* = 22)		
	Pre	Post	Pre-post changes* (*p* = .016)	Pre	Post	Pre-post changes (*p* = .653)
Absent	2	2	(0)	5	3	(− 2)
Mild	7	12	(+ 5)	9	8	(− 1)
Moderate	8	0	(− 8)	3	6	(+ 3)
Severe	3	6	(+ 3)	6	5	(− 1)

^*^Statistically significant *p* < .05. Postintervention lost data: control group (*n* = 1)

### Cervical function

#### Cervical muscle endurance

ANCOVA showed an improvement in cervical muscle endurance in the intervention group (*F* = 5.625; *p* = 0.023). However, this change (Table 4) was not enough to surpass the clinically meaningful threshold of 19.05 s [39]. The ES for the intervention group was large (*d* = 0.72; 95% CI. 09–1.35). No changes in statistical significance were reported when adjusting for covariates.

**Table 4:** Preintervention, postintervention, and change scores for cervical muscle endurance and cervical AROM (*n* = 43)

	Intervention group (*n* = 20)	Control group (*n* = 23)	*p* value*	Cohen’s *d*
DCFET (s) Preintervention Postintervention Pre-post change score	12.17 (6.7) 17.16 (7.35) 5.16 (7.1)	11.05 (7.11) 11.15 (5.67) .10 (6.71)	*p* = .023	.72
Flexion (°) Preintervention Postintervention Pre-post change score	46.26 (13.40) 55.20 (10.45) 8.95 (8.60)†	43.43 (11.87) 41.43 (12.84) − 2.00 (13.90)	*p* = .004	.91
Extension (°) Preintervention Postintervention Pre-post change score	42.45 (13.83) 54.20 (13.02) 11.75 (8.85)†	46.48 (13.17) 41.43 (13.78) − 5.04 (11.78)	*p* < .001	1.57
Affected rotation (°) Preintervention Postintervention Pre-post change score	54.05 (11.52) 64.80 (11.18) 10.75 (11.97)†	51.91 (16.11) 51.57 (13.05) .35 (16.02)	*p* = .015	.76
Unaffected rotation (°) Preintervention Postintervention Pre-post change score	53.70 (15.53) 63.55 (13.30) 9.85 (11.90)†	51.91 (13.13) 51.61 (15.22) − .30 (17.27)	*p* = .033	.66
Affected lateral flexion (°) Preintervention Postintervention Pre-post change score	31.90 (8.78) 39.55 (10.22) 7.65 (7.96)†	32.87 (10.22) 30.26 (10.26) − 2.60 (10.08)	*p* = .001	1.1
Unaffected lateral flexion (°) Preintervention Postintervention Pre-post change score	27.30 (8.70) 37.55 (10.52) 10.25 (5.68)†	29.74 (8.90) 28.26 (10.22) − 1.48 (9.87)	*p* < .001	1.4

Values are mean (SD). The mean difference (SD) for pre-post change score. *Time x group interaction (ANCOVA analysis). ° degrees, *ANCOVA* analysis of covariance, *AROM* active range of motion, *DCFET* deep cervical flexor endurance test, *s* seconds. ^†^*p* < .01

#### Cervical AROM

All cervical AROM variables showed a significant improvement in the intervention group after the ANCOVA (Table 4): flexion (*F* = 9.290; *p* = 0.004); extension (*F* = 27.206; *p* < 0.001); affected rotation (*F* = 6.453; *p* = 0.015); unaffected rotation (*F* = 4.886; *p* = 0.033); affected lateral flexion (*F* = 13.410; *p* = 0.001) and unaffected lateral flexion (*F* = 21.863; *p* < 0.001). All AROM changes in the intervention group were clinically meaningful, as they surpassed the established threshold of 3.6–6.5° [30]. The ES of the MIT intervention was moderate to large depending on the movements: flexion (*d* = 0.91; 95% CI: 0.29–1.54); extension (*d* = 1.57; 95% CI: 0.88–2.25); affected rotation (*d* = 0.76; 95% CI: 0.14–1.38); unaffected rotation (*d* = 0.66; 95% CI: 0.05–1.28); affected lateral flexion (*d* = 1.1; 95% CI: 0.46–1.74) and unaffected lateral flexion (*d* = 1.4; 95% CI: 0.73–2.07). No changes were observed when adjusting for covariates.

### Shoulder AROM

ANCOVA showed a positive significant influence of MIT on the intervention group for the affected abduction (*F* = 11.730; *p* = 0.001), unaffected flexion (*F* = 7.936; *p* = 0.007), and unaffected external rotation (*F* = 5.334; *p* = 0.026), whereas this interaction was not significant for the affected flexion, affected external rotation and both affected and unaffected internal rotation (all *p* > 0.05) (Table 5). The threshold indicating a clinically meaningful change (14–24°) [40] was surpassed for the affected abduction. The ES of the MIT intervention was large for the affected abduction (*d* = 1.3; 95% CI 0.64–1.97) and unaffected flexion (*d* = 0.85; 95% CI 0.22–1.47) and moderate for the unaffected external rotation (*d* = 0.69; 95% CI 0.08–1.31). When adjusting for the tumor stage, changes in unaffected abduction after the MIT protocol were statistically significant (*F*: 4.333; *p* = 0.044), and its ES was moderate (*d* = 0.58; 95% CI − 0.03–1.20).

**Table 5:** Preintervention, postintervention, and change scores for shoulder AROM in degrees (*n* = 43)

	Intervention group (*n* = 20)	Control group (*n* = 23)	*p* value*	Cohen’s *d*
Affected Flexion Preintervention Postintervention Pre-post change score	154.05 ± 11.36 167 ± 9.98 12.95 ± 10.93†	152.41 ± 19.43 154.45 ± 10.7 2.04 ± 17.13	*p* = .245	N/A
Unaffected Flexion Preintervention Postintervention Pre-post change score	154.25 ± 17.49 164.25 ± 12.21 10 ± 11.09†	156.09 ± 21.34 146.61 ± 31.18 − 9.48 ± 29.10	*p* = .007	.85
Affected abduction Preintervention Postintervention Pre-post change score	145.9 ± 26.75 173.75 ± 17.16 26.85 ± 24.02†	148.64 ± 24.90 145.91 ± 24.25 − 2.73 ± 20.60	*p* = .001	1.3
Unaffected abduction Preintervention Postintervention Pre-post change score	155.85 ± 29.60 169.40 ± 18.90 13.55 ± 27.07	150.22 ± 28.21 147.83 ± 34.96 − 2.39 ± 26.50	*p* = .044	.58
Affected external rotation Preintervention Postintervention Pre-post change score	76.60 ± 12.61 84.65 ± 17.24 8.05 ± 13.87	74.04 ± 18.79 74.91 ± 19.51 .87 ± 17.90	*p* = .154	N/A
Unaffected external rotation Preintervention Postintervention Pre-post change score	76.95 ± 15.83 85.30 ± 9.50 8.35 ± 11.26†	75.26 ± 15.8 74.74 ± 18.13 − .52 ± 13.58	*p* = .026	.69
Affected internal rotation Preintervention Postintervention Pre-post change score	73.95 ± 12.21 76.85 ± 10.61 2.9 ± 16.70	71.26 ± 13.40 70.74 ± 11.07 − .52 ± 14.87	*p* = .481	N/A
Unaffected internal rotation Preintervention Postintervention Pre-post change score	73.55 ± 14.39 76.85 ± 12.01 3.3 ± 17.20	71.22 ± 18.85 72.30 ± 15.81 1.08 ± 19.31	*p* = .696	N/A

Values are mean (SD). The mean difference (SD) for pre-post change score. *Time x group interaction (ANCOVA analysis). *ANCOVA* analysis of covariance, *AROM* active range of motion, *N/A* not applicable. ^†^*p* < .01

### Muscular strength and physical fitness

#### Handgrip strength

ANCOVA did not show any significant interaction for either the affected (*F* = 0.342; *p* = 0.565) or unaffected handgrip (*F* = 0.014; *p* = 0.906). Pre- and postintervention values and pre-post-change scores are shown in Supplementary Information 2.1. No changes were observed when adjusting for covariates.

#### IFIS

The chi-squared test did not show any statistically significant changes in the perception of physical fitness evaluated with the IFIS questionnaire on any of its subscales (all *p* > 0.05) (Supplementary Information 2.2).

## Discussion

The objectives of this study were to evaluate the effects of an MIT protocol on mouth opening, TMD, and cervical and shoulder function in the sHNC. In addition, the effects of this protocol on muscular strength and perceived physical fitness were also analyzed. Our results show that a 6-week protocol of MIT improves mouth opening and the perception of TMD, cervical function, and some shoulder AROMs (e.g., flexion, abduction, and external rotation). Moreover, the ES on these variables ranged from moderate to large. However, this protocol does not improve muscular strength or perceived physical fitness in the sHNC.

The MIT techniques used during this RCT were chosen after observing which regions were affected the most in terms of mobility and function after the medical treatment usually provided to the sHNC [5]. The presence of physical impairments of myofascial origin in the sHNC [41] suggests that MIT, as shown in our results, may be an effective treatment to improve some of the previously mentioned sequelae: mouth opening, cervical endurance and AROM, and shoulder AROM.

Mouth opening increased significantly after treatment by MIT. These results agree with those concluded in a systematic review [42]. This increase in mouth opening, which was above the 35 mm stated for the presence of trismus [4] in some of the participants in the MIT group, may be mainly due to the inclusion of both external and intraoral techniques, which allowed the musculature involved in mouth opening to be completely covered. Despite this improvement, the participants in the MIT group did not reach the threshold of 35 mm to consider the absence of trismus; a longer protocol or more active-based techniques could have obtained better results. These types of techniques were also used in other RCTs [43, 44]; however, they were used on patients with TMD, not specifically on sHNC. Therefore, it is necessary to continue performing studies with a similar methodology to demonstrate the long-term effects of MIT on sHNC. As previously stated, the TMD perceived by sHNC, which was also improved in 25% of the participants receiving our MIT protocol, is not the only limiting factor for mouth opening but mainly the fibrosis due to the effects of radiotherapy in both the musculature and connective tissue involved in this function [23].

Our study also demonstrates the positive effects of MIT on increasing cervical muscle endurance, as shown in the DCFET results. This improvement by MIT has already been stated in patients with pain related to mechanical changes [45] and as a short-term improvement in sHNC when applying a protocol of MIT [11]. Our study confirms the benefits of MIT in this population in the short term. However, this interpretation may be taken with caution, as this improvement was not enough to reach the minimal detectable change established to be clinically meaningful [39]: a longer protocol or the inclusion of specific techniques to improve muscle endurance, such as active techniques, may be needed to improve these results.

MIT also showed an increase in the cervical AROM in all its movements and in the shoulder region, specifically flexion (both sides), abduction (affected side), and external rotation (unaffected side). Moreover, this increase was clinically meaningful for the cervical AROM in all its movements; but it was clinically meaningful only for the shoulder abduction on the affected side. This may be since many of the techniques on the MIT protocol were focused on the cervical region, as sHNC presents more impairments at this region [5]. Likewise, MIT has demonstrated a positive effect on shoulder ROM in patients with breast cancer, both in its immediate application and after 4 weeks of treatment [17, 18]. A recently published crossover study demonstrated the positive effects of MIT in cervical AROM in sHNC after a single-session application [11]. However, to the best of our knowledge, our results are the first to demonstrate the effects of a RCT of MIT in sHNC in terms of improvement of AROM. Although the regions of tumor localization and curative medical treatment vary between patients diagnosed with HNC and patients diagnosed with breast cancer, the limiting factors of AROM have a common etiology, such as surgery and radiotherapy [46]; it is then logical to think that in both populations, MIT is an effective treatment to improve these sequelae. If the structural changes induced by medical treatment in patients with HNC produce cervical muscle dysfunction [5], it is possible that MIT and the mechanical stimulation of the connective tissue within its application influence these changes, ameliorating both cervical muscle endurance and AROM.

However, no changes were observed in handgrip strength or in the perception of physical fitness. Grip strength is dependent on skeletal muscle mass, and the loss of both is considered a negative prognostic factor, as it is strongly associated with cancer-related fatigue and poor QoL in patients with HNC [8]. To date, although MIT facilitates the recovery of the function of the locomotor apparatus [14], no studies have demonstrated its effects on these outcomes. It is possible that other approaches focused on the musculoskeletal structures and the physical condition, are needed to change physical fitness in the sHNC.

The current work has some limitations. First, we only evaluated the outcomes after the finalization of the treatment, but it is unknown if these effects remained weeks or months after the end of the treatment. This limitation was due to the pandemic situation originated by the COVID-19, as we could not perform the 1-month follow-up with the lockdown restrictions established in Spain in most of the participants. Second, the same physiotherapist performed all treatments, whereas experiments with different practitioners might help to determine the applicability of MIT in a more similar setting to clinical practice. Moreover, we could not access some clinical data of the participants, and some participants were not able to complete the outcomes assessment; thus, these lost data may have altered the results obtained on these outcomes. In contrast, due to the high response rate of the sHNC, we surpassed the sample size calculated for this study, although this size may not be large enough to reduce interindividual differences, neither to reach clinically meaningful results on all the evaluated outcomes. These limitations may be resolved by performing studies with larger sample sizes and different physical therapists to clarify the effects of MIT in sHNC and assessing the outcomes evaluated in the mid- and long-term after finishing the treatment. Therefore, multimodal approaches may be interesting to develop to deal with systemic impairments such as the decrease in the perceived physical fitness in sHNC.

## Conclusion

A 6-week MIT protocol improves mouth opening, TMD, cervical function, affected shoulder abduction, and unaffected shoulder flexion and external rotation in the sHNC. Clinically meaningful changes were only observed at the cervical AROM and the shoulder abduction. No changes were observed in most of the shoulder AROM, muscular strength, or perceived physical fitness. Future studies should perform longer follow-up designs, increase the sample size, and include multimodal treatments to address these sequelae in the sHNC.

## Supplementary Information

Below is the link to the electronic supplementary material.

Supplementary file1 (PDF 260 KB)

Supplementary file2 (PDF 76 KB)
