# Supplementary material for: The effects of myofascial induction therapy in survivors of head and neck cancer: a randomized, controlled clinical trial
Source: Support Care Cancer. 2022 Dec 17;31(1):49. doi: 10.1007/s00520-022-07482-9 (PMC9758021; doi:10.1007/s00520-022-07482-9)
Supplement: Supplementary file 1 — Supplementary file1 (PDF 260 KB) [file 520_2022_7482_MOESM1_ESM.pdf]

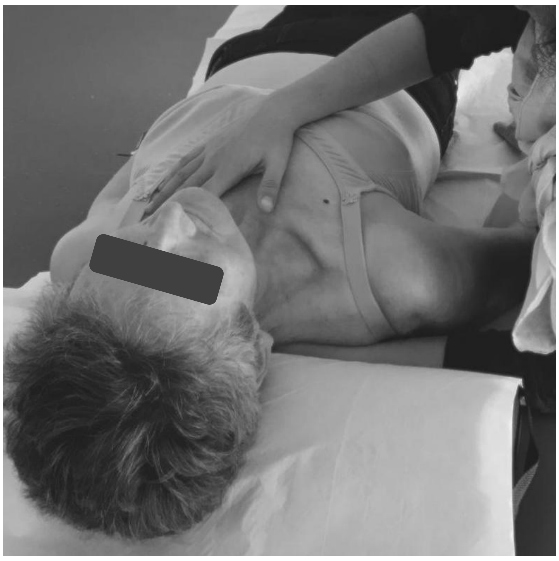

a) Subclavicular technique

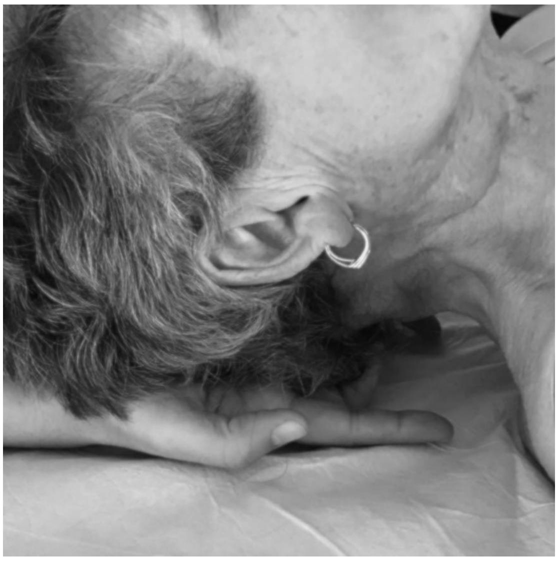

b) Suboccipital Inhibition Release

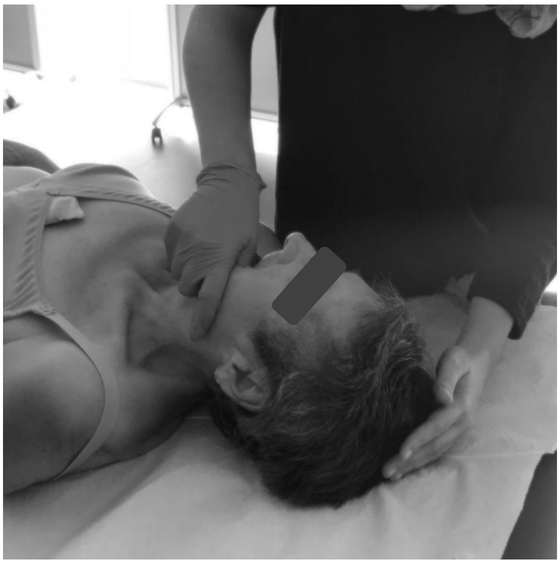

c) Myofascial unwinding over the temporomandibular area

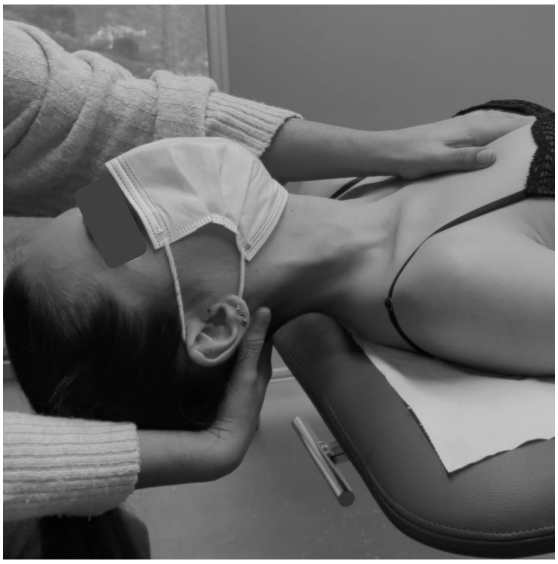

d) Myofascial unwinding of the neck area

Supplementary Information 1 (a-d). Myofascial Induction Therapy techniques applied during the intervention
