# Supplementary material for: The effects of myofascial induction therapy in survivors of head and neck cancer: a randomized, controlled clinical trial
Source: Support Care Cancer. 2022 Dec 17;31(1):49. doi: 10.1007/s00520-022-07482-9 (PMC9758021; doi:10.1007/s00520-022-07482-9)
Supplement: Supplementary file 2 — Supplementary file2 (PDF 76 KB) [file 520_2022_7482_MOESM2_ESM.pdf]

Supplementary Information 2.1. Preintervention, postintervention and change scores for handgrip strength in kg (n=43)

	Intervention Group (n=20)	Control Group (n=23)	*p* value*
**Affected Handgrip (kg)**			
Preintervention	31.65 (15.35)	29.56 (10.9)	
Postintervention	29.7 (11.54)	31.73 (8.67)	
Pre-post change score	-.82 (7.02)	.69 (5.68)	*p*=.565
**Unaffected Handgrip (kg)**			
Preintervention	29.64 (12.27)	45.67 (77.65)	
Postintervention	28.03 (11.44)	32.19 (10.93)	
Pre-post change score	.26 (4.73)	.02 (5.36)	*p*=.906

Values are mean (SD). The mean difference (SD) for pre-post change score. *Time x group interaction (ANCOVA analysis). ANCOVA: Analysis of covariance; Kg: kilograms.

Supplementary Information 2.2. IFIS scores in pre and post evaluations and change scores (n=43)

	Intervention Group (n=20)				Control Group (n=23)			
	Pre	Post	Pre-post changes	p value	Pre	Post	Pre-post changes	p value
**General**				p=.563				p=.340
Very bad	1	1	(0)		0	0	(0)	
Bad	4	6	(+2)		6	4	(-2)	
Adequate	9	6	(-3)		11	8	(-3)	
Good	0	1	(+1)		5	0	(-5)	
Very good	1	0	(-1)		1	1	(0)	
**Cardiorrespiratory**				p=.446				p=.364
Very bad	3	2	(-1)		2	3	(+1)	
Bad	7	7	(0)		6	6	(0)	
Adequate	3	5	(+2)		10	3	(-7)	
Good	2	0	(-2)		4	1	(-3)	
Very good	0	0	(0)		1	0	(-1)	
**Strength**				p=.622				p=.629
Very bad	0	1	(+1)		1	0	(-1)	
Bad	6	4	(-2)		8	5	(-3)	
Adequate	8	7	(-1)		11	8	(-3)	
Good	1	2	(+1)		2	0	(-2)	
Very good	0	0	(0)		1	0	(-1)	
**Speed**				p=.223				p=.557
Very bad	2	0	(-2)		1	1	(0)	
Bad	5	7	(+2)		7	5	(-2)	
Adequate	6	7	(+1)		12	5	(-7)	
Good	2	0	(-2)		1	2	(+1)	
Very good	0	0	(0)		2	0	(-2)	
**Flexibility**				p=.802				p=.427
Very bad	1	1	(0)		0	1	(-1)	
Bad	10	7	(-3)		13	9	(-4)	
Adequate	3	5	(+2)		7	3	(-4)	
Good	1	1	(0)		2	0	(-2)	
Very good	0	0	(0)		1	0	(-1)	

Pre intervention lost data: Intervention group (n=5); Post intervention lost data: Intervention group (n=6); control group (n=10) IFIS: International Fitness Scale
